# Detection and Characterization of Circulating Tumor Cells Using Imaging Flow Cytometry—A Perspective Study

**DOI:** 10.3390/cancers14174178

**Published:** 2022-08-29

**Authors:** Anna Muchlińska, Julia Smentoch, Anna J. Żaczek, Natalia Bednarz-Knoll

**Affiliations:** Laboratory of Translational Oncology, Medical University of Gdańsk, 80-210 Gdańsk, Poland

**Keywords:** liquid biopsy, circulating tumor cells, imaging flow cytometry

## Abstract

**Simple Summary:**

Liquid biopsy is non-invasive approach used to prognose and monitor tumor progression based on the detection and examination of metastasis-related events found in the patients’ blood (such as circulating tumor cells (CTCs), extracellular vesicles, and circulating nucleic acids). Different ultrasensitive techniques are applied to study those events and the biology of tumor dissemination, which in the future might complement standard diagnostics. Here, we suggest that CTCs analysis could be improved by the usage of imaging flow cytometry, combining advantages of both standard flow cytometry (high-scale analysis) and microscopy (high resolution) to investigate detailed features of those cells. From this perspective, we discuss the potential of this technology in the CTC field and present representative images of CTCs from breast and prostate cancer patients analyzed with this method.

**Abstract:**

Tumor dissemination is one of the most-investigated steps of tumor progression, which in recent decades led to the rapid development of liquid biopsy aiming to analyze circulating tumor cells (CTCs), extracellular vesicles (EVs), and circulating nucleic acids in order to precisely diagnose and monitor cancer patients. Flow cytometry was considered as a method to detect CTCs; however, due to the lack of verification of the investigated cells’ identity, this method failed to reach clinical utility. Meanwhile, imaging flow cytometry combining the sensitivity and high throughput of flow cytometry and image-based detailed analysis through a high-resolution microscope might open a new avenue in CTC technologies and provide an open-platform system alternative to CellSearch^®^, which is still the only gold standard in this field. Hereby, we shortly review the studies on the usage of flow cytometry in CTC identification and present our own representative images of CTCs envisioned by imaging flow cytometry providing rationale that this novel technology might be a good tool for studying tumor dissemination, and, if combined with a high CTC yield enrichment method, could upgrade CTC-based diagnostics.

## 1. Liquid Biopsy

Liquid biopsy is the non-invasive alternative to traditional diagnostic tools that could provide complementary information about tumor aggressiveness and even allow monitoring of patients’ outcomes in real time during and/or after treatment. It has been developed intensively during the last two decades and consequently became a clinically relevant alternative diagnostic tool. Liquid biopsy aims to investigate circulating tumor cells (CTCs), extracellular vesicles, or circulating nucleic acids in different body fluids with an unchangeable main focus on blood samples. An ultrasensitive methodology has been developed in order to identify and analyze those rare signatures of tumor progression or resistance to therapy at the potential early stages of the disease [1]. Historically, the first described liquid-biopsy-related events in tumor patients were CTCs [2]. Since then, the presence of CTCs was proven to identify patients with poor prognosis in many solid tumors, as well as predict tumor progression or responses to therapy [1]. Multiple technologies have been applied to isolate and enrich those rare cells and subsequently characterize them. However, today, the only FDA-approved systems are the CellSearch^®^ system (Menarini Silicon Biosystems, Castel Maggiore, Italy) and the newly cleared Parsortix^®^ system (Angle plc, Surrey, UK) dedicated to CTC isolation combined with detection or exclusive isolation, respectively. Nevertheless, low yields of CTCs and epithelial markers dependence remain a bottleneck of this field, despite the intense efforts to improve these technologies [3,4]. Thus, novel sensitive and specific methods allowing identification of a wider spectrum of heterogenous populations of disseminating cells are urgently needed. It is particularly crucial as, apart from CTCs, progression-relevant cells such as circulating fibroblasts, macrophages, or endothelial cells isolated from blood have been considered to improve stratification of tumor patients and deepen knowledge of biology of metastatic spread [5,6,7,8].

## 2. Flow Cytometry in Liquid Biopsy

Flow cytometry (FC) is a high-throughput, sensitive technique that allows fast quantitative detection of multiple antigens simultaneously. Most common FC instruments usually offer 5–12 detectors that can measure different fluorescently labeled antigens in addition to forward- (FSC) and side-scatter (SSC), describing size and granularity, respectively. Thus, usage of FC in liquid biopsy could substantially improve characterization of CTCs and hypothetically could even enable the sorting of those rare cells for further molecular analysis or culturing.

Several studies have proven that FC might indeed be feasible for the detection of rare cells, and its sensitivity is similar or higher than real-time PCR analysis [9,10]. However, there are large discrepancies between studies: 1 cell of interest may be detected among 10^4^–10^6^ other cells [11,12,13,14]. The comparative study on different CTC-detecting techniques showed that the recovery rate using FC in spiked cell experiments might be similar compared to CellSearch^®^ (i.e., 74–85% vs. 41–88%) [15,16,17,18]. It is also supposed to be less biased by interoperate variance in comparison to non-automated methods, such as manual microscopy. In the examined solid tumors (i.e., colorectal, breast, and ovarian cancer), sensitivity and specificity of CTC detection using FC was similar to CellSearch^®^ (i.e., 50.8%–90.16% and 77.42%–97%, vs. 54% and 95%, respectively) [18,19,20] or even higher (e.g., even 6.5-fold) [21].

Prior to FC-based CTC detection, the most frequently applied procedure to isolate CTCs was a buffy coat isolation by Ficoll/Histopaque density gradient centrifugation [21,22] with or without red blood cell lysis [20,23]. Sample-enrichment methods were used very rarely [23]**,** and Bahnassy et al. showed that they did not really impact CTC counts obtained by FC [18]. Most studies using FC in CTC detection were performed in blood samples from colorectal cancer patients with standard epithelial markers, such as EpCAM [22,24], K20 [25], K5/6/8/17 [26], and EpCAM/pan-K [18,27] used for CTC identification. Recently, this type of analysis has also been improved by incorporation of vimentin [23], CD133, CD54, and CD44 [28], or PTEN and androgen-receptor splice variant 7 [29] staining in colon/breast, colorectal, and prostate cancers, respectively. CTC presence was observed in 71%–91% of patients depending on the cut-off value for CTC counts (i.e., from ≥1 to ≥5 detected CTCs) and status of metastatic disease [24,25,26,27]. CTCs identified using FC are described to be clinically relevant [24,28,29,30]. Nevertheless, the reported frequency of CTC-positive patients is, in general, relatively high in comparison to other studies using different technologies. The study of Coumans et al. [21], with higher CTC yields detected by FC rather than CellSearch^®^, could potentially emphasize significant problems for the application of FC technology in this particular field, i.e., potential overestimation of CTC number, especially in patients with low number of CTCs/mL of blood. It has to be considered that despite many advantages of using FC in the detection of rare cells, FC does not assure visual confirmation of cell identity, which makes it technically challenging to distinguish genuine CTCs from false positive events. In addition, conventional FC does not provide brightfield, whereas cell morphology might be an important criterion to identify a cell as a CTC. All in all, a relatively high number of detected events combined with lack of their exact status visual verification could explain why this method has never been used on a large scale to identify CTCs.

## 3. Imaging Flow Cytometry—Pros and Cons in CTC Field

### 3.1. General Description

Combining flow cytometry with high-resolution microscopy brought an upgrade of flow cytometry, creating genuine opportunity to implement this method into CTC technologies [31]. Imaging flow cytometry (imFC) was introduced to the market in 2011. Since then, it has been widely used for detection of extracellular vesicles [32,33,34], whereas, to the best of our knowledge, only limited studies have focused on CTC detection and characterization using this method [35,36,37,38,39]. This novel method combines high-throughput flow cytometry and detailed imaging of high-resolution microscopy, resulting in an open-platform which is useful for the fast detection of multiple markers of interest in an unlimited number of cells in suspension [40].

Indeed, imFC enables fast acquisition of images of all the cells in an investigated sample (high-scale analysis), their selection is based on multiple features and gating strategies, as well as subsequent manual verification similar to the one applied during CellSearch^®^ analysis [41]. Notably, sensitivity and specificity of CTC detection using imFC are in principle similar to FC [35,39,42,43,44,45]. However, the keystone of this technology in this context is its potential for the experimenter to verify the cells, which significantly improves the final outcome, typical for FC. Acquisition of 1.5 mln PBMCs in six fluorescent channels lasts approx. 30 min, and—according to the manufacturer—up to 75 features per channel might be considered during subsequent analysis (for example, they are useful in CTCs detection: size, staining intensity and localization, roundness of an examined cell). Thus, standard and even upgraded criteria for CTCs selection might be applied and performed in the standardized way. Duration of selection and verification of CTCs from a collected population of all cells varies between patients and depends on the applied gating strategy but lasts usually up to a couple of hours. Taking into account its speed and quality, imFC might successfully compete on the market of imaging devices for CTC detection. To some extent, it might be even used as an alternative to the CellSearch^®^ system, offering higher magnification and more detailed images than so far used as the gold standard (20–60× vs. 10× magnification).

Notably, the estimated total price of the analysis also seems to be much lower (even 7.5×) using imFC [35]. As immunofluorescent staining procedures applied for imFC are fully open platforms dependent only on the unique experiment design, the experimenter is flexible to choose any antibody and antibody’s source/provider, which could also anticipate the lowering of the total price of analysis per sample. In combination with a high CTC yield enrichment method, it would potentially lower the cost significantly and exceed the quality of outcomes obtained using CellSearch^®^ system.

### 3.2. Detailed Analysis and Standardization

**Figure 1:**
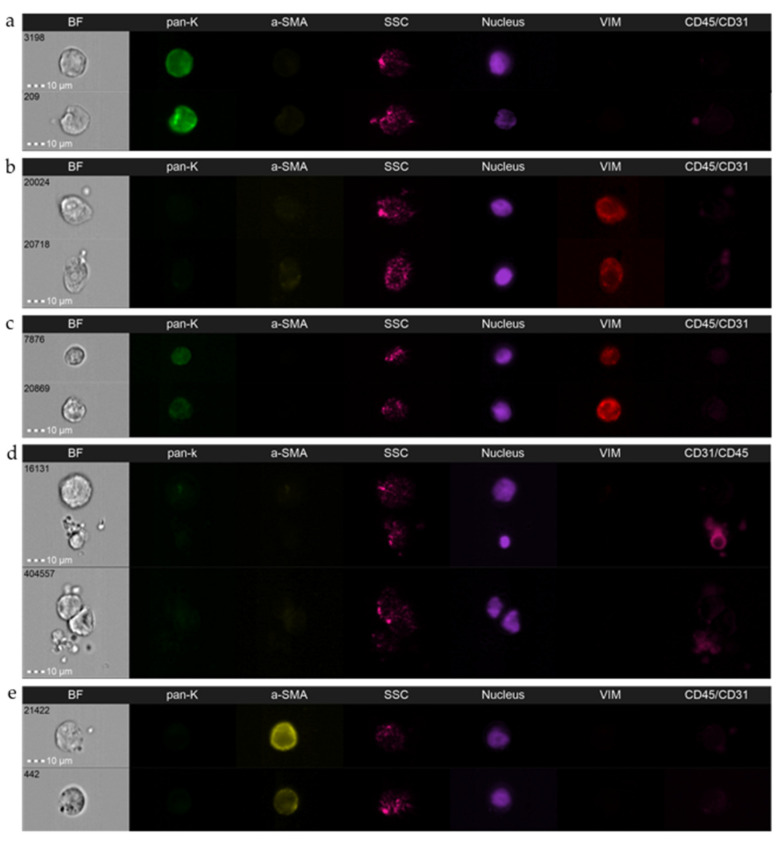
Representative pictures of the spectrum of epithelial-to-mesenchymal phenotypes in circulating tumor cells are detected using imaging flow cytometry in breast cancer patients: (**a**) epithelial CTC, (**b**) mesenchymal CTC, (**c**) epithelial–mesenchymal CTC, (**d**) double negative CTC, and (**e**) circulating cancer-associated fibroblasts. BF—brightfield; pan-K—pan-keratins; a-SMA—alpha-smooth muscle actin; SSC—side scatter; Vim—vimentin; CD45/CD31—leukocyte/endothelial cell marker. Amnis^®^ ImageStream^®^X Mk II (Luminex, Austin, TX, USA), objective 40×.

**Figure 2:**
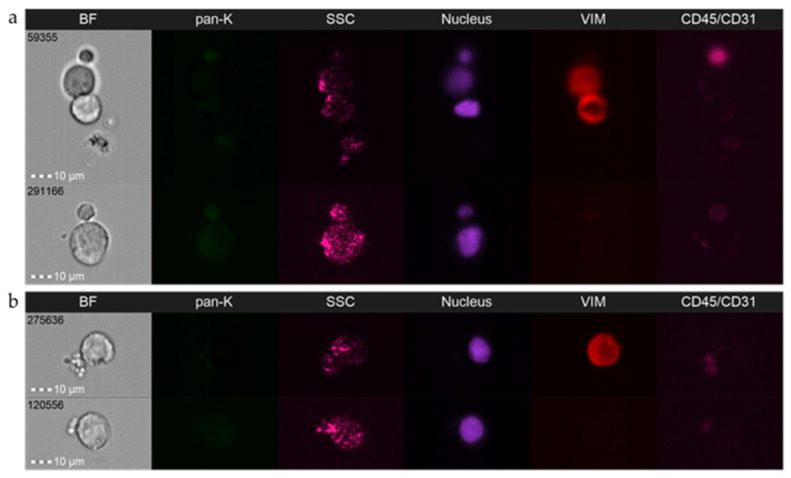
Representative pictures of circulating tumor cells (DAPI(+)CD45/CD31(−)) isolated from prostate cancer patients interacting with (**a**) normal cells (CD45/CD31(+)) and (**b**) platelets performed using imaging flow cytometry. BF—brightfield; pan-K—pan-keratins; SSC—side scatter; Vim—vimentin; CD45/CD31—leukocyte/endothelial cell marker. Amnis^®^ ImageStream^®^X Mk II (Luminex, Austin, TX, USA), objective 40×.

Multichannel platforms enable simultaneous analyses of up to nine fluorescent markers plus cell morphology in the brightfield (2×) and its granularity in the SSC channel. Reserving two channels for the nucleus (e.g., DAPI) and exclusion marker (e.g., leukocyte marker, CD45), seven fluorescent channels might yet be dedicated to detection of putative CTC markers or markers differentiating CTCs and interacting normal cells, or alternatively other subpopulations of circulating progression-relevant normal cells (e.g., circulating fibroblasts, macrophages, or endothelial cells) [46]. Using a multimarker approach is particularly important in this field as it warrants the discrimination of different phenotypes, such as predictive phenotypes or broad spectrum of EMT-related phenotypes (Figure 1a–d) [39], including the phenotypes which were so far invisible to the applied technologies (i.e., DAPI(+)CD45/31(−) epithelial/tumor cell markers(−) cells; Figure 1d) and facilitates the differentiation of those rare phenotypes from other cells, e.g., circulating cancer-associated fibroblasts (Figure 1e). It also empowers identification of normal interactors of tumor cells, such as leukocytes and platelets or others, based on the exclusively morphological features evaluated in the brightfield channel (Figure 2a,b).

**Figure 3:**
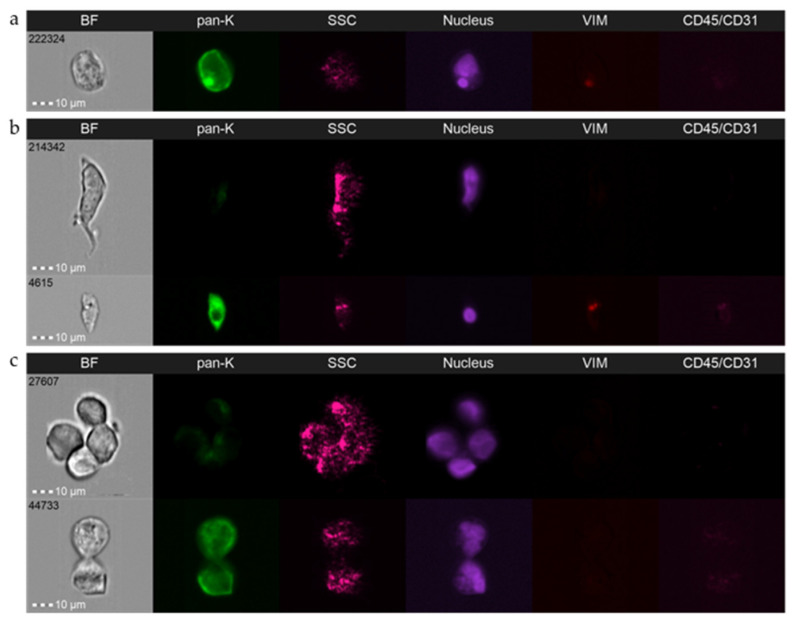
Representative pictures of CTC’s morphological details envisioned by imaging flow cytometry: (**a**) presence of micronucleus; (**b**) cell protrusions; (**c**) clusters stereometry. Amnis^®^ ImageStream^®^X Mk II (Luminex, Austin, TX, USA), objective 40×.

Note that, as imFC benefits from high-resolution imaging characteristics for advanced microscopes, it permits unique detailed morphological analysis of investigated cells, which were vastly limited in CellSearch^®^. Depending on the device equipment (20×, 40× and/or 60× objectives), imFC magnification exceeds roughly the 2–6-times magnification offered by CellSearch^®^. Cells are snapshotted in flow; however, imFC produces a large proportion of images with sharp focus (incl. two images in brightfield), where morphological details of investigated cells are visible and measurable even in lower magnifications (Figure S1). Evaluation of such images might provide unique information on CTCs morphology so far lost in previous analyses, e.g., presence of protrusions and structural details of the nucleus, such as micronucleus and stereometry of CTC clusters (Figure 3) [47]. So far, few studies have shown the significance of such parameters in characterization of CTCs and their clinical relevance [48,49], whereas this new technology could potentially validate those observations. Such advanced technology could potentially also improve the criteria of CTC definition and in turn, fine-tune CTC diagnostics in the future.

It is worth stating the fact that imFC creates unique opportunity for genuine standardization of CTC detection, which is a long-awaited holy grail in this field [50]. All parameters of acquisition and gating are fully adjustable and controllable (e.g., sample’s flow speed, power of lasers, cut-off of intensity of fluorescence background), and might be performed in an identical manner to dissect interpatient differences. This approach could potentially also improve the evaluation of gradual expression of some proteins, including therapeutic targets, such as hormone receptors or HER2.

### 3.3. Caveats and Challenges

In sum, the method has a lot of advantages resulting from a cross of standard flow cytometry and advanced microscopy, and might be easily adapted to CTCs detection. Nevertheless, some caveats and challenges need to be addressed.

The method itself generates undefined cell loss (by usage of approx. 3–6 µL of cell suspension for standard precalibration of the flow parameters for each sample), whereas it would be crucial to work with the initially high numbers of cells or at least highly enriched populations of cells. Thus, the combination of imFC with a high-yield-enrichment method (such as leukapheresis) seems to be a necessary yet intricate issue. CTCs are rare events, ergo samples need to be potentially highly enriched to find the needle in the haystack. On the other hand, the staining of cells in suspension generates loss of cells even after the reduction of staining procedures to minimal steps and are followed by an aforementioned unmeasurable loss of cells at the precalibration step of imFC acquisition. In that sense, the imFC procedure requires a significant number of input cells and eliminates methods such as MACS-based enrichment or others that significantly limit the total number of cells.

This problem is also combined with another technical issue occurring during usage of imFC analysis, i.e., the compromise between data storage and acquisition. The imFC allows the acquisition of all cells in a sample, which could potentially improve our understanding of metastatic spread and exceed analyses to non-CTC progression-related objects, even from the previously analyzed samples. As a generated file size relies mostly on a number of used fluorescent channels, magnification, and the number of documented cells’ images (i.e., gating strategy), data collected from the DAPI(+) gate (i.e., all analyzed cells in sample) are a few times larger than those from the DAPI(+) CD45(−) gate (e.g., average raw files has the capacity of 30–50 GB vs. 5–8 GB per sample if analyzing ca. 1 mln input cells using 40× magnification and six fluorescent channels). It generates significant storage and processing problems. Large files take hours to process (by using compensation matrix and analysis template overlay), while smaller files are much easier to work with, but lose additional information on normal or atypical cells. Thus, gating strategy is the key step into the CTCs experiment setup with all positive or negative consequences. Decisions on this compromise must be addressed in future analyses.

The method allows detailed morphological characterization of the analyzed cells. From our study on the epithelial–mesenchymal spectrum of phenotypes of CTCs in 196 and 98 blood samples obtained from breast and prostate cancer patients, respectively (data not shown), we assume that CTCs criteria need to be carefully revised and adjusted to specific cancer (sub)types. New parameters such as stringent criteria on morphology of nucleus and/or nucleus-to-cytoplasm ratio should be included in CTCs defining processes in order to reduce the risk of false-positive results. Indeed, atypical objects occur frequently during imFC analyses (e.g., pan-K(+)/CD45(+) cells, cells of <4 µm diameter, large pan-K(+)/CD45(−) cells with small nuclei, etc.), and need to be classified carefully in separate sets of objects. They appear with different frequencies between patients, thus they merit careful further investigation in order to improve general CTC identification and deepen analysis aiming to define their putative relevance in tumor progression [51,52,53,54].

The device also allows the fluidics speed to be chosen, but higher speed involves more blurred photos. Therefore, obtaining the best image quality can take more time (approximately 30 min for 30 μL of suspension containing approx. 1.5 mln cells). In general, after analysis of over 2000 CTC in total at the magnification of 40× and low speed, we observed the minority of the cells investigated by imFC to be out of focus. Nevertheless, CTCs cannot be relocated manually to reassure their staining details as it happens in fluorescent microscopy. A lack of manual observation of individual selected objects in different planes makes the analysis of CTC clusters difficult, but this is not really the case for single CTCs, as cellular morphology is still depicted at a high resolution in all fluorescent channels and two brightfield channels (with different focuses). If a study is designed in the correct way, taking into account fluorescent channels crosstalk and separation of fluorochromes for detection of proteins with similar cellular localization, in our opinion, the obtained images will be sufficient for the classification of the investigated cells and will significantly exceed the quality of CellSearch^®^. It is important to mention here that, in theory, it is possible to use nine fluorescent channels for imFC, but designing and optimizing experiments with that number of target proteins coexisting in CTCs or even separated between two different populations of cells (e.g., CTCs and circulating endothelial cells or circulating fibroblasts) is rather complicated and limited by several factors, which are also typical for fluorescent microscopy. Similar cellular localization of examined proteins might significantly influence the number of co-investigated markers, whereas finding several good-quality fluorochromes with a signal that will not be visible in the adjacent channel is difficult. Applied in imFC, compensation is then useful; however, even careful application in the case of CTCs detection is challenging and might be only a suboptimal solution.

Last but not least, the image quality and biological aspects of investigated objects are the issues which might be linked with the next challenge for the CTC field, i.e., implementation of machine learning in CTCs identification. As already mentioned, imFC facilitates the collection of the images of all cells in an analyzed fraction. Such huge library of normal cells vs. CTCs can potentially support an introduction of artificial intelligence methods to discriminate between those cells [55,56,57,58,59]. Finally, imFC with its high magnification option (i.e., 60×) might potentially allow the deepening of CTCs characterization by the detection of genetic aberrations assessed by fluorescent in situ hybridization (FISH) alone or in combination with protein markers [60,61,62]. The feasibility of FISH performed on cells in suspension has been already shown in leukemia by optimization of so called immuno-flowFISH [63]; however, validation of its robustness in rare cells still needs investigation.

## 4. Conclusions

Imaging flow cytometry opens a new avenue in liquid biopsy. It is an open platform enabling the multitarget phenotyping of CTCs independently of epithelial markers and enables the multiparametric morphological evaluation which has been unreachable with technologies used so far. In combination with good CTC-enrichment methods, it has the potential to significantly improve liquid biopsy and knowledge on tumor dissemination.

## Supplementary Materials

The following supporting information can be downloaded at: https://www.mdpi.com/article/10.3390/cancers14174178/s1, Figure S1: Representative pictures of morphological details of leukocytes isolated from blood of healthy donors and envisioned by different objectives of imaging flow cytometry.
