# Supplementary material for: Detection and Characterization of Circulating Tumor Cells Using Imaging Flow Cytometry—A Perspective Study
Source: Cancers (Basel). 2022 Aug 29;14(17):4178. doi: 10.3390/cancers14174178 (PMC9454939; doi:10.3390/cancers14174178)
Supplement: Supplementary file 1 [file cancers-14-04178-s001.zip › cancers-1814838-supplementary.pdf]

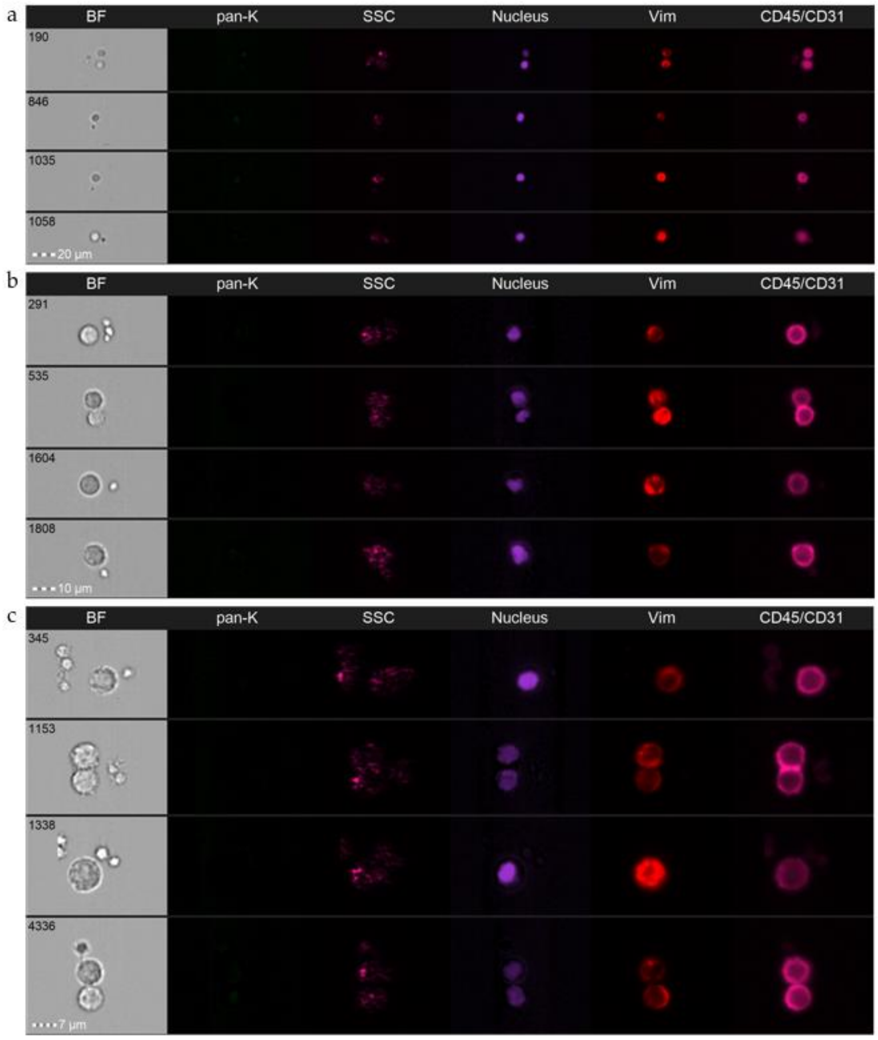

Figure S1. Representative pictures of morphological details of leukocytes isolated from blood of healthy donors and envisioned by different objectives of imaging flow cytometry: (**a**) 20×, (**b**) 40×, and (**c**) 60×. Amnis® ImageStream®X Mk II (Luminex).
